# The Building Blocks of Child Bilingual Code-Mixing: A Cross-Corpus Traceback Approach

**DOI:** 10.3389/fpsyg.2021.682838

**Published:** 2021-07-27

**Authors:** Antje Endesfelder Quick, Stefan Hartmann

**Affiliations:** ^1^Faculty of Philology, Institute of British Studies, University of Leipzig, Leipzig, Germany; ^2^Faculty of Arts and Humanities, German Department, University of Düsseldorf, Düsseldorf, Germany

**Keywords:** corpus linguistics, traceback method, usage-based linguistics, code-mixing, individual differences

## Abstract

This paper offers an inductive, exploratory study on the role of input and individual differences in the early code-mixing of bilingual children. Drawing on data from two German-English bilingual children, aged 2–4, we use the traceback method to check whether their code-mixed utterances can be accounted for with the help of constructional patterns that can be found in their monolingual data and/or in their caregivers' input. In addition, we apply the traceback method to check whether the patterns used by one child can also be found in the input of the other child. Results show that patterns found in the code-mixed utterances could be traced back to the input the children receive, suggesting that children extract lexical knowledge from their environment. Additionally, tracing back patterns within each child was more successful than tracing back to the other child's corpus, indicating that each child has their own set of patterns which depends very much on their individual input. As such, these findings can shed new light on the interplay of the two developing grammars in bilingual children and their individual differences.

## Introduction

Usage-based accounts of linguistic phenomena have become increasingly important in the field of language acquisition. In this paper, we apply this account to bilingual language acquisition, and more specifically to utterance-internal child bilingual code-mixing, i.e., the simultaneous use of two languages within one utterance, e.g., ***aber bloß****a little bit* “but just a little bit” (Silvie, 3;07)^1^. Code-mixing, or code-switching^2^, has been a recurrent topic both in sociolinguistics and in psycholinguistics (see Bullock and Toribio, 2009; Gardner-Chloros, 2009 for an overview). Most research on code-mixing so far (e.g., Poplack, 1980; Myers-Scotton, 1997; MacSwan, 2000; Cantone, 2007) has adopted a structuralist/constraint-based framework. Both formalist and usage-based approaches are of course more heterogeneous in terms of theory and methodology than this simplified, coarse-grained division might suggest, and there is also a considerable degree of overlap between them (see e.g., Yang, 2016). As a general tendency, however, it seems fair to say that most formalist proposals focus more on structural constraints on code-mixing, while usage-based accounts are more interested in the cognitive mechanisms that underlie code-mixing. From a usage-based perspective, it can be assumed that the same mechanisms that have been shown to drive monolingual acquisition play a role in multilingual acquisition and code-mixing as well. These mechanisms include analogy and pattern finding (Tomasello, 2009) as well as chunking and frequency effects (see e.g., Diessel, 2019).

Recent studies have therefore started to investigate code-mixing from a usage-based perspective (e.g., Quick et al., 2018; Vihman, 2018). In this study, we follow up on this trend, focusing on the role of multi-word patterns as well as on individual differences between speakers. According to the usage-based approach to language acquisition, children acquire their linguistic knowledge based on their experience with the world and what they hear (e.g., Tomasello, 2003). Since no two children live the same life and each has different input situations, it stands to reason that variation in the output is the norm and each child has their own inventory of constructions. Monolingual children already exhibit an enormous range of variation. In multilingual speakers, variation is likely to be greater by virtue of being exposed to two languages and their respective interlocutors who speak different languages in different contexts. Multilingual speakers will frequently produce code-mixed utterances such as ***der moon kann****fly* “the moon can fly” (Fion, 3;2.12). In this paper, we discuss how bilingual code-mixing can be assessed in an exploratory, data-driven way, taking individual differences into account. To do so, we compare the code-mixing of two German-English bilingual children. First, we give a brief overview of the usage-based perspective on (monolingual as well as multilingual) language acquisition and code-mixing before we turn to our corpus-based case study, in which we inductively analyze the language use of two bilingual children on the basis of longitudinal corpus data.

## Theoretical Preliminaries and Main Hypotheses

The usage-based approach to language acquisition assumes that children acquire language by finding patterns in the input they receive (for an overview see Ambridge and Lieven, 2011; Ibbotson, 2020). Given the assumption that children structure their first words and utterances around their immediate experiences, it follows that their inventory of linguistic knowledge does not solely consist of words and grammar. Rather, the inventory is a mixture of lexically specific units (single words like *cat, dog*, as well as multiword expressions like *what's this?*) and frame-and-slot patterns like [*what's* X?] (see e.g., Tomasello, 2003: pp. 105–108). Multi-word units (MWUs), i.e., sequences of frequently co-occurring words, play a particularly important role in language acquisition. They can be acquired in different ways: Either the MWU is always encountered as such by the child and therefore not segmented and acquired as a whole, or the MWU emerges gradually through the frequent co-occurrence of certain words. But no matter how a unit was formed, it does not always have to stay a unit: Over time, children tend to break up multi-word units, e.g., using them as the basis for frame-and-slot patterns by opening a variable slot, and thus arriving at a more productive use of their language (see e.g., Ambridge and Lieven, 2011: pp. 133–136). Consequently, the composition of inventories changes constantly and any description will always be a snapshot. Nevertheless, these snapshots are important because they tell us something about the ways children process and acquire language, as well as about the interplay of language and cognition.

Bilingual children are of particular interest because they can show patterns in their speech that are different from monolingual speech, such as code-mixing or other transfer phenomena (Koch and Günther, 2021). As mentioned in section Introduction, a large body of research has been accumulated (e.g., MacSwan, 2000; Bernardini and Schlyter, 2004; Cantone, 2007). While previous studies have acknowledged the existence of individual differences and distinguished different types of code-mixing, they mainly concentrated on the categorization of the various types of mixing and on describing constraints on code-mixing, linking them to potential underlying principles (e.g., Di Sciullo et al., 1986; Myers-Scotton and Jake, 2015). However, various studies have shown that these constraints are tendencies at best which very often cannot accommodate counterexamples, and have called for more flexible and dynamic models (e.g., Vihman, 2018; Backus, 2021).

Recently, a set of studies has taken code-mixing onto usage-based grounds suggesting that fixed chunks and frame-and-slot patterns that have been shown to play a major role in monolingual acquisition can also account for children's code-mixing. Lexically fixed patterns make execution faster and less effortful because they are uttered without close monitoring (e.g., Havron and Arnon, 2021). On this view, code-mixing is suggested to be constructed around frame-and-slot patterns with the frame activated in one language and the open slot being filled with elements from the other language, e.g., [*that's my __*] + *Bademantel* “bathing gown” → *that's my* ***Bademantel***(Fion, 03;11.16). Quick et al. (2019) have shown that many of the patterns attested in one child's code-mixing could be traced back to the caregivers' input, suggesting that the child extracted patterns from the input to use it in his code-mixing. If we now compare the language of two children and their respective input, we should find individual differences in their use of patterns and their inventories. These differences can be expected to project into their code-mixing: Children make use of different patterns in their code-mixing. Code-mixing in bilingual children offers us a window into the complexities and interplay of the two developing grammars, which is why we will focus on code-mixed utterances in our corpus study to account for the individual inventories of the two children under scrutiny.

In order to do so, we need a reliable method to identify these inventories. In previous work, we have shown that the traceback method established by Lieven et al. (2003, 2009) and Dabrowska and Lieven (2005) for analyzing monolingual data is well-suited for identifying recurrent patterns in multilingual data as well (Quick et al., 2019, 2021). The basic idea of this method is to trace back all utterances in a test corpus to previous utterances based on a limited set of operations (see below for details). The traceback method was initially developed to substantiate the hypothesis that early child language is highly formulaic and organized around a very limited set of “pivot schemas.” Indeed, the proportion of successful tracebacks proved to be very high consistently across all traceback studies. This in turn lends support to the position that children learn language from the input they receive, without any need for an innate “language acquisition device.” These results could be obtained across different languages, including German (Koch, 2019) and Italian (Miorelli, 2017), although it should be stressed that the way in which the traceback method operationalizes the detection of constructions works best in languages with a relatively fixed word order (see Miorelli, 2017; Koch, 2019; also see section Conclusion below). The method has also been used as a starting point for a more in-depth analysis of the constructional patterns that it retrieves. When studying code-mixing, the method can give clues to what extent children draw on frame-and-slot patterns that can also be found in their monolingual data, as well as in the input they receive. Thus, the use of the traceback method serves multiple complementary goals: Firstly, it allows us to quantify the extent to which a child's early language use can be accounted for with a relatively simple set of fixed chunks and frame-and-slot patterns. Secondly, it allows us to identify those patterns, which also allows us to characterize each child's individual inventory of constructions. This in turn can give clues as to the individual differences between children. Thirdly, the traceback method allows us to check to what extent the patterns in a child's output overlap with patterns attested in the input they receive.

In this paper, we extend a previous study by Quick et al. (2019) by discussing what the traceback method can contribute to inductively identifying individual differences in children's code-mixing. Our aim is to check (a) whether the code-mixed utterances can be constructed from the monolingual ones and (b) how much each child's output correlates with the caregivers' input. In addition, (c) we cross-correlate each child's output with the input and the monolingual language use of the other child. Our main hypothesis is that there is a high degree of overlap between the patterns identified in the individual children's language use, including their code-mixing, and those identified in their caregivers' data. In addition, we expect that the proportion of successful tracebacks will be smaller for the cross-corpus than for the within-corpus studies due to the individual differences between the children and their input situation.

## Corpus Study

### Participants

For the present study, we investigate two German-English bilingual children, Silvie and Fion. Both children grew up in one-parent-one-language (OPOL) households with one parent being a native speaker of English and the other being a native speaker of German. Both children lived in Germany, came from a middle-class household and are simultaneous German-English bilinguals. The two children were not acquainted with each other.

The first child, Silvie, had an English-speaking mother and a German-speaking father. The father's proficiency in English was very limited and the parents therefore spoke German to one another. The corpus covers recordings from 2;4 until 3;10 years of age, averaging to about 2.5 h of recordings per week. For our analyses we included a total of 37,995 child utterances and 193,993 caregiver utterances.

Fion is the second child to a German-speaking mother and an English-speaking father. Although the parents mostly adhered to the OPOL strategy when they talked to Fion, they did not settle on a family language and sometimes used both languages interchangeably when all family members were present. Fion's data covered a span from 2;3 to 3;11 and 47,812 child utterances as well as 180,293 input utterances entered the analyses. The input utterances include a small amount of data from Fion's older brother, who also grew up as a simultaneous bilingual and sometimes used code-mixing when talking to Fion or his parents. The data were transcribed and enriched with a small set of annotations by the first author. For non-standard word forms, normalized forms were annotated (e.g., *gasbet* > *basket, de muffin* > *the muffin*, etc.). These normalized forms were also used for the traceback analysis.

### Corpus Analysis

To analyze the corpus data, we draw on the traceback method, which allows us to identify recurrent patterns in the data and which will be described in detail in the following section. In discussing the results, we additionally draw on an exploratory analysis of word pairs (bigrams) attested in the code-mixed data. Taken together, these approaches can help us detect the “building blocks” of code-mixed utterances in the early speech of bilinguals.

#### The Traceback Method

We follow Quick et al. (2019), who used a variant of the traceback method established in seminal works like Lieven et al. (2003) and Dabrowska and Lieven (2005). In the traditional application of the method, a longitudinal corpus documenting the language acquisition of one child is split into two parts: the test corpus, which usually contains the last two recording sessions, and the main corpus, which contains all previous recordings. The goal of the method is to show that the vast majority of the child's utterances in the test corpus have predecessors in the main corpus, i.e., they are either verbatim repetitions (called “fixed strings”), or they can be accounted for with the help of “templates” that are partially lexically specific and contain an open slot, such as [*I want* REFERENT]. In addition, the method can help us answer the question which patterns the child uses.

Quick et al. (2019) deviate from the traditional application in the way they carve up their dataset into main and test corpus: Investigating the code-mixing of Fion, they use the child's code-mixed data as test corpus and the child's own as well as his caregivers' monolingual utterances as main corpus. In this way, they show that even most of the child's code-mixing can be accounted for on the basis of partially filled constructions. This suggests that in essence, the same patterns that account for children's monolingual language use can also account for their code-mixing. In the present study, we extend this analysis, combining it with a cross-corpus traceback approach as proposed in Koch et al. (2020). While we only relied on utterance-initial *n*-grams in Quick et al. (2019), the computational implementation of the traceback algorithm in this paper is closer to the original traceback method, although it is still simplified in order to allow for a fully automatic analysis^3^. For the present study, we used an algorithm that works as follows (see data availability statement for more detailed information):

1. For each utterance in the test corpus, it checks whether there is a verbatim match in the main corpus. If there is a match, the derivation is considered successful.
2. If there is no match in the main corpus, it checks whether a frame-and-slot pattern can account for the utterance. To do so, up to two consecutive words are replaced by a wildcard in the search expression (equivalent to the SUBSTITUTE operation in the classic traceback procedure). For example, if our target utterance is ***das hat****time out* “this has time out” (Fion, 02;03.12), the algorithm will check if attestations of *__ hat time out, das __ time out, das hat ___ out, das hat time __, das hat __, __ time out, das __ out* are attested in the corpus at least twice (the threshold established by Dabrowska and Lieven, 2005). Then the algorithm checks if the omitted words (e.g., *das* in the case of __ *hat time out*) are attested in the main corpus. Only if this is the case, the pattern candidate is treated as valid. If there are multiple valid pattern candidates, the ones with the longest consecutive fixed string are preferred, e.g., *das hat __* (two consecutive words in the fixed string) is preferred over *das __ out* (only one word before and after the open slot). Also, pattern candidates with utterance-initial fixed strings are preferred over candidates with an utterance-initial open slot: Given the tendency toward right-headedness in both English and German and given the results of earlier studies (see e.g., Cameron-Faulkner et al., 2003 on the relevance of utterance-initial elements in child-directed speech), this promises more plausible results. However, the rule of longest consecutive strings is prioritized over the rule to prefer utterance-initial patterns. If no pattern candidate fulfills the requirements (at least two occurrences in the main corpus, and the omitted words have to be attested in the main corpus as well), then the derivation is considered unsuccessful.

We used the code-mixed utterances (*N* = 3,501 for Fion and 4,279 for Silvie) as test corpus and (a) the child's own monolingual utterances and (b) the caregivers' data as main corpora. In a second step, we used (c) the *other* child's monolingual data and (d) the *other* child's input as the main corpus. We refer to (a) and (b) as within-corpus traceback and to (c) and (d) as cross-corpus traceback. Using the within-corpus approach, we check to what extent the children's code-mixed utterances can be accounted for with the help of fixed chunks and frame-and-slot patterns that they have either used themselves or that they have heard in their input. The cross-corpus approach can help us to get a clearer impression of the extent to which the linguistic repertoires of the two children differ. Compared to other implementations of the traceback method, our approach entails the disadvantage that the pattern detection process does not take semantic and/or syntactic information into account, which can lead to rather implausible patterns being postulated. However, there is no guarantee that the linguistically informed patterns identified in previous traceback studies are psychologically plausible (see e.g., Hartmann et al., 2021). The purely data-driven approach can also be seen as an advantage as it detects patterns purely on the surface level without making far-reaching a-priori assumptions.

#### Results

**Figure 1:**
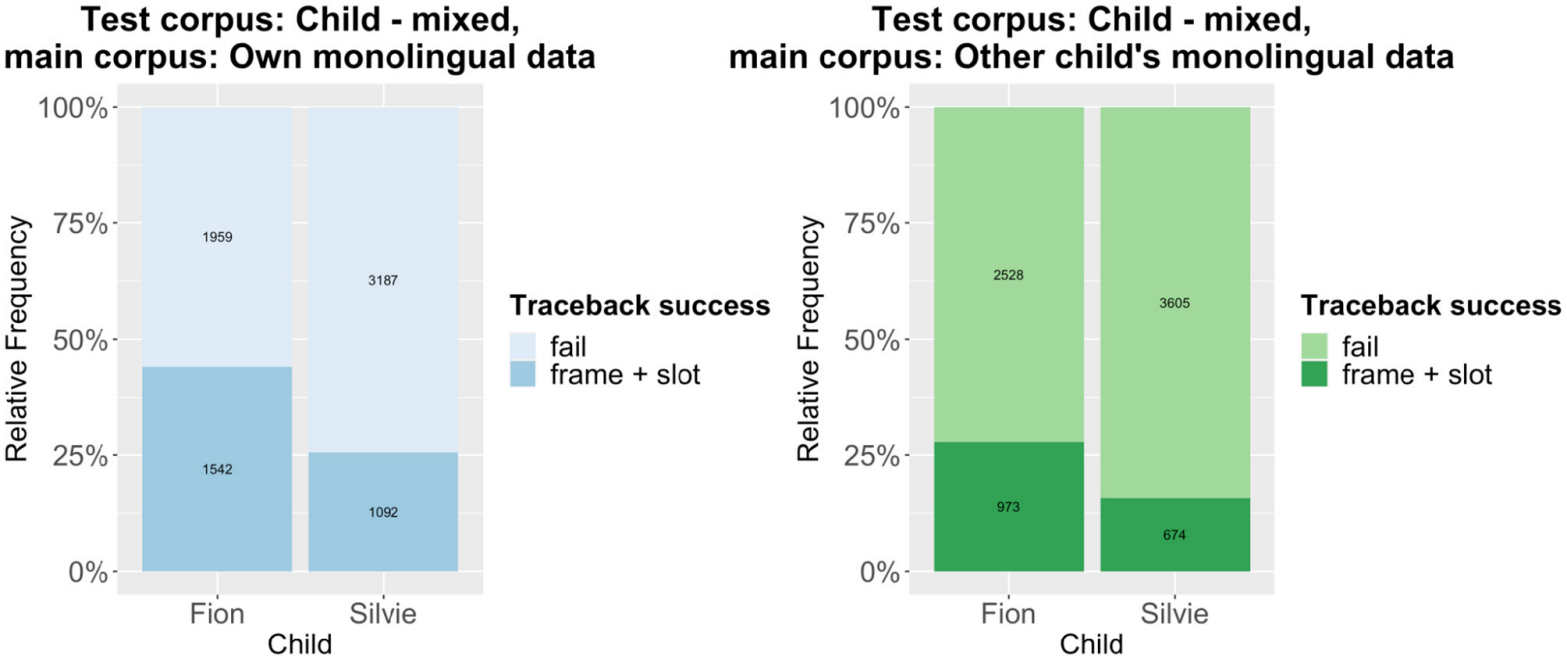
Traceback results: Code-mixed data to monolingual data.

**Figure 2:**
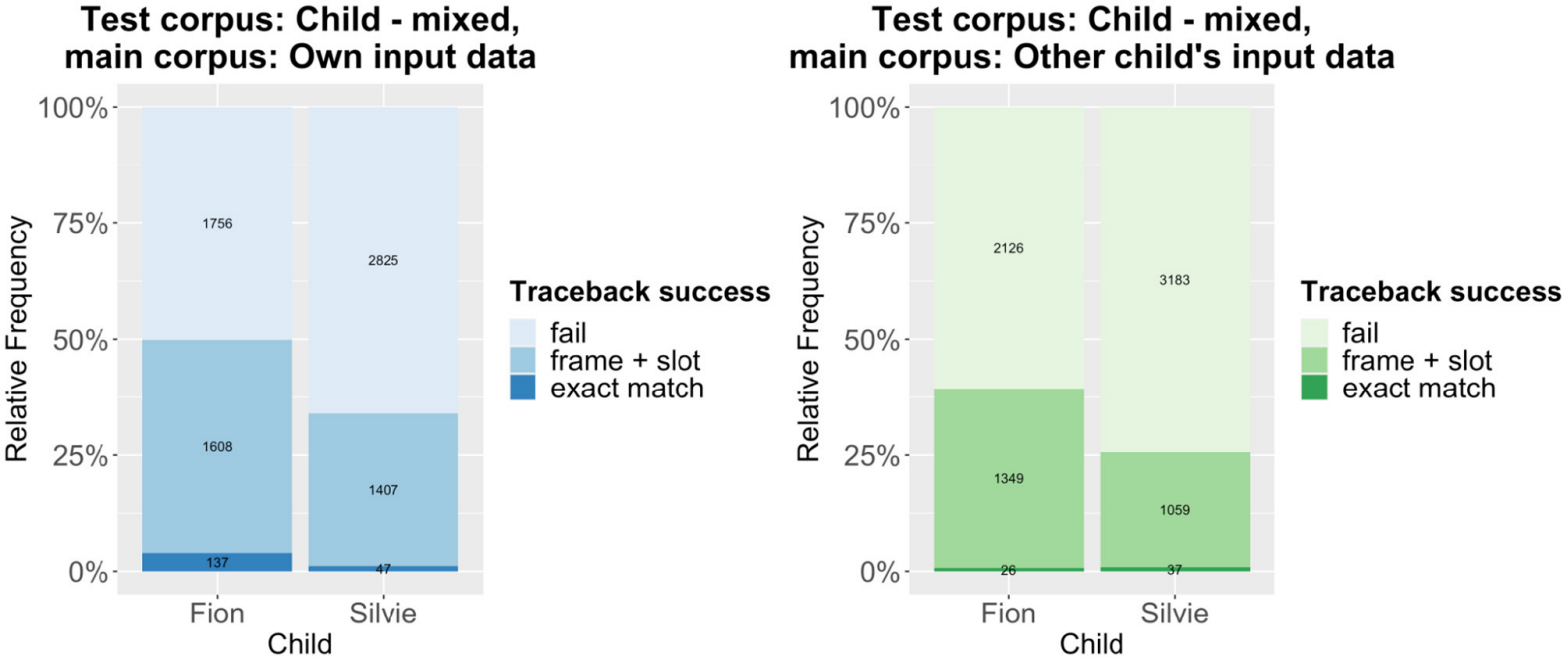
Traceback results–code-mixed data to input data.

The traceback results are summarized in Figures 1, 2. Figure 1 shows the results of tracing the code-mixed data to the monolingual data, while Figure 2 shows the results that are obtained when using the caregivers' input as main corpus. Compared to other traceback studies, the success rate is comparatively low. However, we have to remember that the test corpora only include code-mixed utterances, while the main corpora almost exclusively consist of monolingual utterances (except for very few code-mixed utterances in the caregivers' input; the children's own monolingual data of course only contain monolingual data). Given that the traceback method can be considered a quite conservative approach (see e.g., Quick et al., 2021), it is actually quite remarkable that about 50% of all utterances can be successfully derived (in the case of Fion). As expected, the traceback success is much lower for the cross-corpus results, both when tracing the patterns detected in the code-mixed data to the input and when tracing them to the monolingual data. A mixed-effects logistic regression model using traceback success as the response variable, traceback type (within-corpus vs. cross-corpus) as the predictor variable, and the utterance as random effect shows that the difference is highly significant across both corpora, regardless of whether the child's own monolingual data or the caregivers' input is used as main corpus (Table 1).

**Table 1:** Coefficients of mixed-effects logistic regression models fit to the traceback data.

	**Estimate**	**Std. Error**	**z value**	**Pr(>|z|)**
**Code-mixed data** **→** **Monolingual data Monolingual data**				
**Fion**				
(Intercept)	−8.8	0.22	−40	0^***^
Type: cross-corpus	−7.6	0.27	−28	5.20E−170^***^
**Silvie**				
(Intercept)	−9.2	0.21	−43	0^***^
Type: cross-corpus	−6.3	0.3	−21	4.70E−99^***^
**Code-mixed data** **→** **Input data**				
**Fion**				
(Intercept)	−8.3	0.22	−37	2.80E−305^***^
Type: cross-corpus	−4.5	0.32	−14	6.50E−45^***^
**Silvie**				
(Intercept)	−9.5	0.23	−42	0^***^
Type: cross-corpus	−6.2	0.46	−13	1.00E−40^***^
**All child utterances** **→** **Input data**				
**Fion**				
(Intercept)	1.57	0.051	3.076E + 01	1.0E−207^***^
Type: cross-corpus	−1.20E + 00	0.034	−3.600E + 01	7.5E−281^***^
**Silvie**				
(Intercept)	−0.18	0.048	−3.70	2.0E−04^***^
Type: cross-corpus	−1.30	0.035	−36.00	1.6E−287^***^

*** *p < 0.001*.

**Figure 3:**
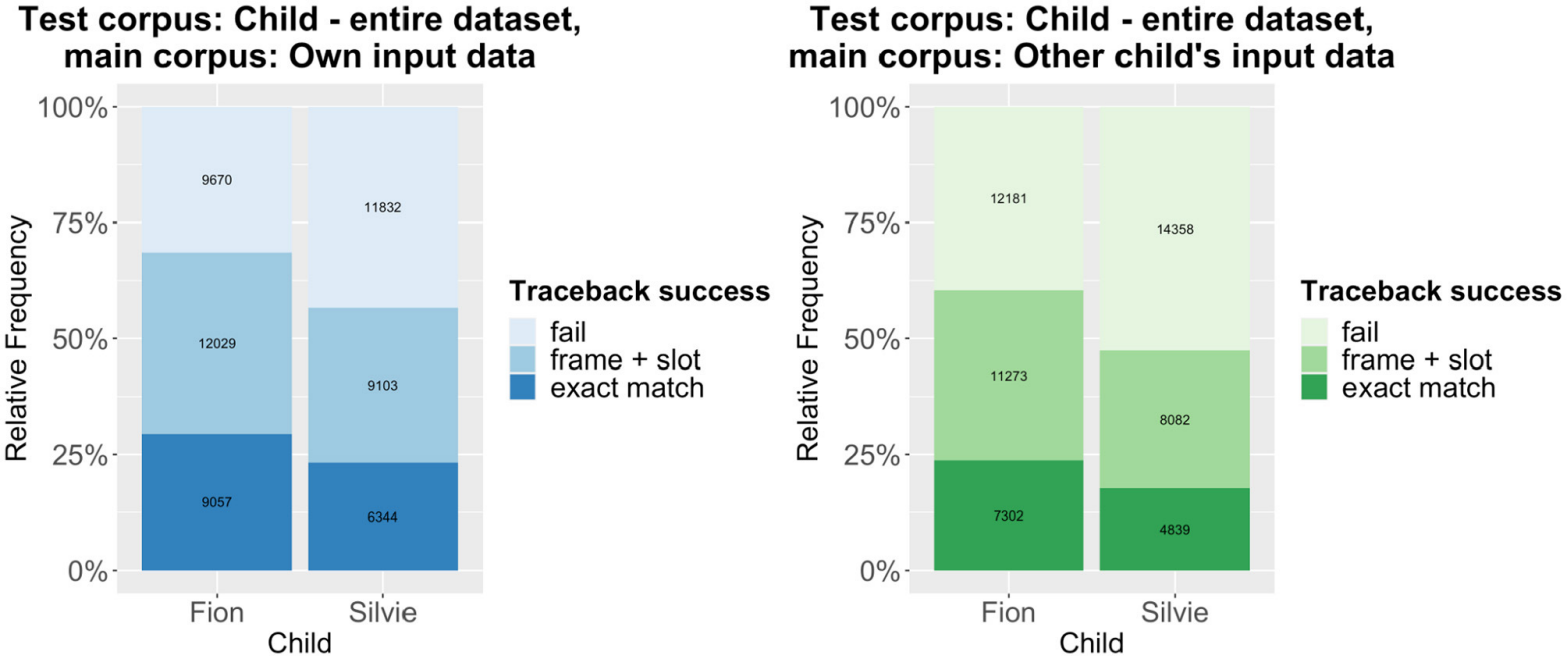
Traceback results using all utterances of each child as test corpus and the caregivers' utterances (left) or the other child's caregivers' utterances (right) as main corpus.

Thus, our prediction that there are significant differences in traceback success between the within-corpus and the cross-corpus approach is confirmed. That being said, there is still much overlap between the results of both approaches, which indicates that a substantial number of patterns are shared between the two children. The individual differences between the two children rather become clear in another aspect of the results: Across the board, the traceback success for Silvie is much lower than for Fion. This also holds if we use each child's entire dataset as test corpus, as shown in Figure 3 (note that the overall traceback success is much higher if the monolingual utterances are taken into account). Again, the differences are highly significant according to a binomial mixed-effects regression model (Table 1).

The difference in traceback success between the two children is in line with the previous studies mentioned above, which have shown that Silvie's language development is, overall, more advanced than Fion's. As her utterances are longer and her grammar is more complex, the traceback method will inevitably produce more fails.

While the proportions of successful and failed tracebacks can be highly instructive, it can only be the first step of any traceback study. When investigating code-mixed utterances, it can be particularly revealing to take a closer look at the patterns detected by the traceback algorithm. Table 2 shows the most frequently attested patterns for each child. Again, it has to be emphasized that our exploratory use of the traceback method claims no cognitive plausibility of the detected patterns but only serves as a proof-of-concept that the code-mixed utterances can, in principle, be accounted for using frame-and-slot patterns. Both children tend to combine *this* with German material—in the case of Fion, this pattern even accounts for no <100 utterances. Apart from that, the patterns are relatively similar, and they substantiate the usage-based assumption that most instances of code-mixing can be accounted for with the help of simple frame-and-slot patterns. As a very rough tendency, the “frames” of *wh*-questions and simple assertive sentences (***ich bin kein***“I am no”) tend to come from German in both Fion's and Silvie's case, while frequently attested content words like *fire, water, cheese* come from English.

**Table 2:** Twenty most frequently attested patterns detected by the traceback method.

**Pattern**	**Freq_Fion**	**Pattern**	**Freq_Silvie**
__ this	100	__ this	24
__ **da**	20	__ this one	20
**noch mehr** __	16	**ein** __	14
__ that	15	__ cheese	11
**ein** __	11	**und** __	11
**ich will** __	11	**das ist** __	10
__ you	11	__ red	9
**und ein** __	10	**nein** __	8
__ cheese	9	__ blue	8
__ **feuer**	9	**noch ein** __	8
__ **nicht**	9	__ green	8
**nein ein** __	8	**du bist** __	8
__ milk	8	**ich bin** __	8
**das istis** __	7	__ water	8
**ich** __	7	**ich bin kein** __	7
this __	7	__ **auch**	6
**was ist** __	7	**ja** __	6
**wo eine** __	7	__ cake	6
**wo istis** __	7	**der** __	6
**wo kommt** __	7	**du bist ein** __	6

*German materials are indicated in bold*.

**Figure 4:**
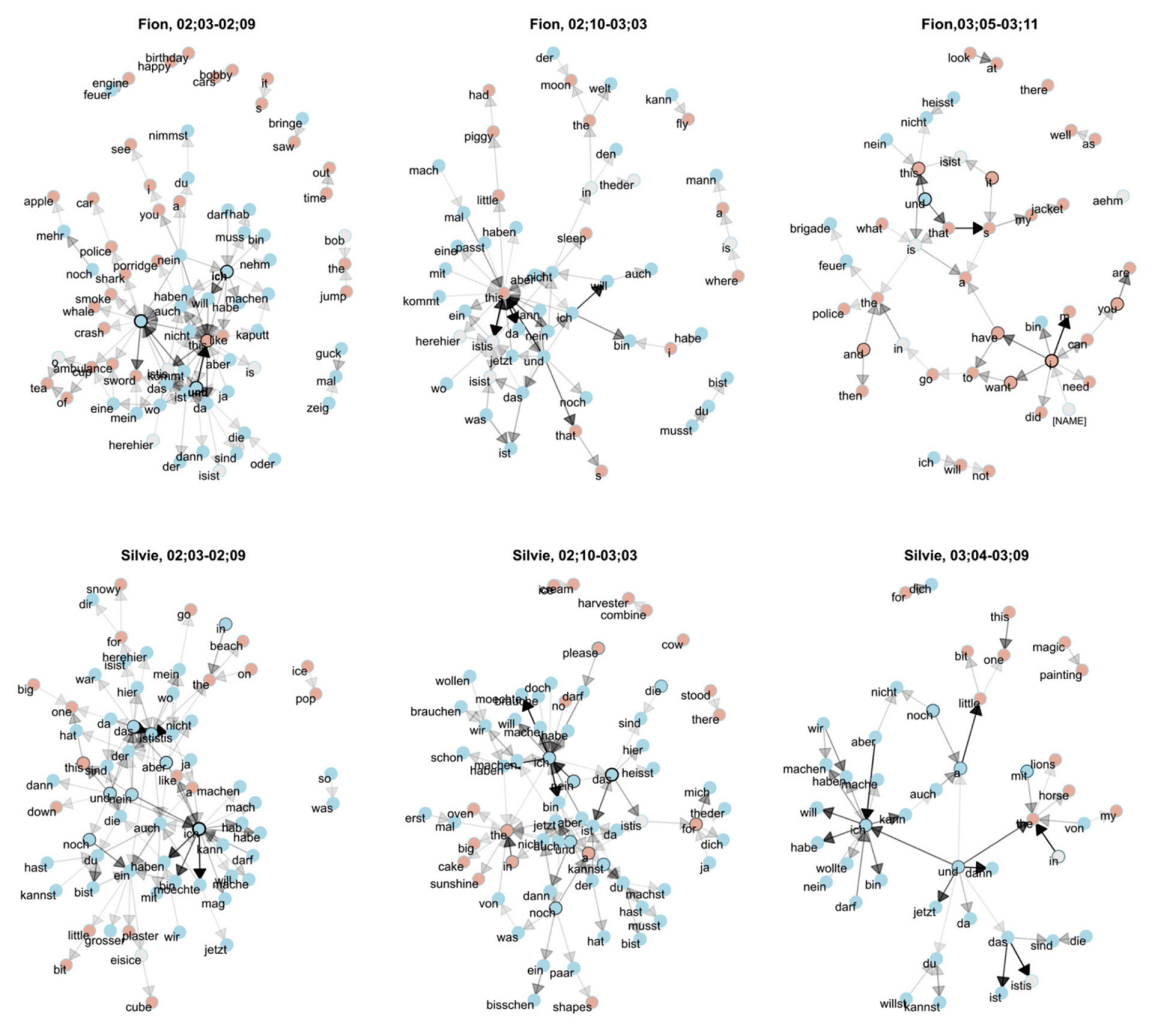
Bigram network based on all code-mixed utterances in the corpus, created using ggraph (Pedersen, 2021).

Note that in all cases, not only are the fixed strings in the frame-and-slot pattern attested in the main corpus (here: the child's own monolingual data) but also the fillers that occur in the open slots in the individual utterances. The fact that a large proportion of code-mixed utterances can be derived successfully with the help of the traceback method lends further support to the usage-based assumption that children's early language use is strongly item-based, i.e., structured around concrete exemplars. This also becomes clear if we take a look at the data from a different but related perspective, by focusing on the word pairs (bigrams) attested in the code-mixed utterances as shown in Figure 4. This figure shows, for each word (type) in the corpus, the immediately succeeding words attested in each child's utterances. The transparency of the arrows indicates the transition probability between words: Highly frequent bigrams are indicated by a black arrow, while rare bigrams are indicated by a transparent (hence, gray-ish) arrow. For instance, in Fion's earliest data depicted in the upper-left panel of the plot, ***und***“and” (highlighted with boldface) is strongly connected to *this*, as indicated by the thick black arrow. This means that the bigram ***und****this* occurs frequently in the data. The other word highlighted in boldface, ***ich***“I,” is often followed by ***will***“want.” It is also fairly often preceded by ***nein*** “no” or ***darf***“may,” but these connections are not as strong as those between ***ich*** and ***will***.

In line with the traceback results, we can see immediately that there are certain “hubs” that combine with many different words from both languages (blue indicating German, red English, and gray words that cannot be assigned unambiguously to one of the languages). Figure 3 also shows a developmental perspective. As can be expected, Silvie's network is initially more complex, which is in line with the finding that her language use is more complex and more creative than Fion's; over time, however, both children's networks grow smaller as their use of code-mixing decreases.

## Conclusion

In this paper, we have taken a usage-based and data-driven approach to bilingual language acquisition. We have used the traceback method to investigate whether the patterns attested in two bilingual children's code-mixing can be traced to their monolingual data and to their caregivers' input, and we have used a cross-corpus traceback approach in order to check to what extent the linguistic repertoires that the two children use in their code-mixing overlap. Results showed that first, code-mixed utterances are often constructed around frame-and-slot patterns, which, second, can be traced back to the monolingual utterances as well as, third, to the input. This is in line with what we expect from a usage-based perspective: The more frequently children use a pattern, the more it becomes entrenched, and the easier it is to activate. Our findings also resonate with studies that show children's uptake from child-directed speech: Parents often use recurrent patterns which in turn are also often used in their children's early production (Cameron-Faulkner et al., 2003).

We also found that the traceback success is significantly lower in the case of the cross-corpus approach, i.e., when tracing utterances from one corpus to the other. This is not surprising as each child has a different input situation from which they extract their individual linguistic knowledge. It is also clear that differences cannot be too large and that speakers need to converge on an inventory that overlaps in order to understand each other. However, there are also considerable individual differences that become clear when we look at the overall traceback success, which is lower for Silvie's data. All in all, Silvie's language is more complex, which also becomes clear in the bigram network that we have used to take a bird's-eye perspective on the children's code-mixing. Both the traceback study and the inspection of the bigram network, however, substantiate our hypothesis that the usage-based theory of language acquisition, according to which children's early utterances are organized around a limited set of concrete items (e.g., Tomasello, 2003), can account not only for monolingual, but also for multilingual language acquisition, and even for code-mixing.

However, the limitations of the traceback method, as discussed in e.g., Hartmann et al. (2021), should be kept in mind. Perhaps most importantly, the traceback method is largely limited to the detection of distributional patterns. As such, it presupposes a construction-centered approach to language (acquisition). As e.g., Wasserscheidt (2020: p. 61) points out, there is a long-standing debate between lexically-oriented and construction-based approaches. From a language acquisition point of view, Behrens (2007: p. 261) presents substantial empirical evidence in favor of “the construction as the primary conveyor of meaning.” However, most usage-based theorists would readily acknowledge that lexical and constructional knowledge interact in language production and comprehension. As such, (syntactic) constructions alone are not enough to fully account for language acquisition. Drawing on an early precursor to the traceback method developed by Lieven et al. (1997), Vihman (1999) has argued that the role of semantic learning should not be underestimated: Her analysis of English-Estonian acquisition data suggest that predicate types and structures play a major role in the process of language learning. The traceback method, however, only identifies fixed strings and frame-and-slot patterns. It can at best provide indirect evidence regarding speakers' knowledge about the properties of individual lexical items. These caveats also apply to the exploratory study of bigrams. It would therefore be worthwhile to complement the inductive approach presented here with follow-up studies that take a different methodological perspective on the same data.

In addition, it should be kept in mind that our analysis was only exploratory, and follow-up studies should adopt a more fine-grained approach to the data. For one thing, adding a morphological annotation layer to the data could help to (semi-)automatically identify recurrent frame-and-slot patterns in a more reliable and psychologically plausible way. In addition, a systematic analysis of the failed tracebacks as conducted in previous traceback studies could add important insights (see e.g., Koch, 2019). Also, while we have only taken intra-utterance code-mixing into account, it would be interesting to investigate (emergent) individual differences in bilingual language acquisition against what is known from studies on individual differences between adult speakers. For example, Street and Dabrowska (2010) have shown that individual differences are more likely for infrequently used constructions, whereas high-frequency syntactic patterns show less variability. Multilingual acquisition data can help us understand how such individual differences in language attainment come about and what role the linguistic input plays in this respect. Another desideratum would be to account for individual differences in bilingual speakers' attainment of the different languages they acquire—after all, it is well-known that bilingual speakers differ in their personal fluencies along various dimensions (see e.g., Edwards, 2013: pp. 11–14). The corpora analyzed in the present paper provide a rich source of data for approaching these and related questions in subsequent studies. In the long run, it would be desirable to extend the approach to other language pairs, which could prove insightful not only from a theoretical but also from a methodological perspective, as it could help to explore whether the traceback method can be fruitfully applied to pairs of typologically very different languages: In the case of German and English, the method works well as the structure of both languages is fairly similar, even though German has a slightly freer word order (see Koch, 2019: p. 212). But for another language, Italian, Miorelli (2017) has shown that the method meets some challenges, which are of course amplified when two languages are involved. The exploratory results presented in this paper can therefore only be a starting point for a broader theoretical and methodological discussion of how code-mixing should be modeled from a usage-based point of view.

## Data Availability Statement

The data used for the present study are not yet publicly available but can be obtained upon request. The code used for data analysis as well as samples of the data are available at https://hartmast.github.io/Quick_Hartmann_2021_Building_Blocks/.

## Ethics Statement

Ethical review and approval was not required for the study on human participants in accordance with the local legislation and institutional requirements. Written informed consent to participate in this study was provided by the participants' legal guardian/next of kin.

## Author Contributions

All authors listed have made a substantial, direct and intellectual contribution to the work, and approved it for publication.

## Conflict of Interest

The authors declare that the research was conducted in the absence of any commercial or financial relationships that could be construed as a potential conflict of interest.

## Publisher's Note

All claims expressed in this article are solely those of the authors and do not necessarily represent those of their affiliated organizations, or those of the publisher, the editors and the reviewers. Any product that may be evaluated in this article, or claim that may be made by its manufacturer, is not guaranteed or endorsed by the publisher.

## Footnotes

^1^Throughout the paper, German material is presented in boldface. All examples from the corpora described below.

^2^While these terms are often used interchangably, we follow Muysken (2000) in using the term “code-mixing” for utterance-internal code-switching.

^3^In future studies, we plan to enrich the corpus with part-of-speech tags to allow for a more fine-grained automatic analysis.
